# Quantifying the effects of human activities and climate variability on runoff changes using variable infiltration capacity model

**DOI:** 10.1371/journal.pone.0272576

**Published:** 2022-09-01

**Authors:** Qingling Bao, Jianli Ding, Lijing Han

**Affiliations:** 1 College of Geography and Remote Sensing Sciences, Xinjiang University, Urumqi, China; 2 Key Laboratory of Smart City and Environment Modelling of Higher Education Institute, Xinjiang University, Urumqi, China; University of Kashmir, INDIA

## Abstract

Detecting and assessing changes in the hydrologic cycle and its response to a changing environment is essential for maintaining regional ecological security and restoring degraded ecosystems. There is no clear scientific evidence on the effects of human activities and climate variability on runoff and its components in typical arid areas. Therefore, in this study, a heuristic segmentation algorithm, a variable infiltration capacity model (VIC), and remote sensing data to quantify the effects of human activities and climate variability on runoff in the catchment of Lake Ebinur, Xinjiang, China. The results found: (1) The heuristic segmentation algorithm divided the study period into reference period (1964–1985) and two impact periods: I (1986–2000) and II (2001–2017). (2) Cropland and forest land showed an increasing trend, with grassland and barren land accounting for most of the increase. At the same time, the leaf area index (LAI) increased by 0.002 per year during the growing season. (3) Compared with the reference period, runoff depth decreased by 108.80 mm in impact period I due to human activities, but increased by 110.5 mm due to climate variability, resulting in an overall increase in runoff depth of 1.72 mm. Runoff depth increased by 11.10 mm in the impact period II compared to the reference period, with climate variability resulting in an increase of 154.40 mm, but human activities resulted in a decrease of 143.30 mm. Our results shed light on decision-making related to water stress in changing circumstances in arid regions.

## 1. Introduction

Climate change and human activities are important drivers of the hydrologic cycle and water management decisions [1]. Climate change, especially temperature rise and changing precipitation patterns, will have a lasting impact on the distribution patterns of regional hydrologic processes in space and time [2]. In the context of global warming, hydroclimatic variables in arid and semi-arid regions have been found to show an opposite trend to those in humid regions, where the climate in the Tarim River basin is changing toward warm and humid [3,4]. In addition, the intensification of human activities in recent decades, such as economic and demographic factors, agricultural reclamation, and urbanization, has led to increased pressure on water resources in arid regions [5]. The Global International Waters Assessment Project (GIWA) water resources assessment for Central Asia found that 70% of economic development conflicts are due to water scarcity [6]. To alleviate the severe ecological and water management problems in the northern region, the Chinese government has initiated more than five ecological restorations programs (ER) since 1978, including the Three Northern Forest Conservation Forests, the Soil and Water Conservation Program, the Partnership Against Desertification, the Return of Cropland to Forests, and the Grassland Ecological Conservation Program [7–9]. However, there is no clear scientific evidence on the impacts of human activities and climate variability on hydrological systems in arid regions.

Determining changes in the regional hydrological cycle is a major challenge due to the complex interactions between hydrological systems and elements such as climate and land cover [10]. Recently, a growing number of studies have separated the impacts of climate change and human activities on hydrological systems using four methods, namely: (a) the traditional Budyko-based approach [11–13]; (b) the trend method of time series decomposition [14]; (c) the scenario-based approach to hydrologic system modeling [15,16]; and (d) the Tomer-Schilling approach [17,18]. Among them, the techniques based on Budyko and the time-trend are currently the most widely used, while the application of the latter two methods is gradually increasing. Zhang, Nan used eight different forms of time trend methods and compared them with the Budyko method to show that human activities have a smaller impact on runoff than climate change [19]. Xie, Liang used simulations of variable infiltration capacity scenarios **(**VIC) to evaluate the impact of ER projects on the regional hydrologic system in northern China [3]. Yan, Bai used the Soil and Water Assessment Tool model (SWAT) to compare the changing state of runoff relative to the baseline period through breakpoint analysis and delineation of impact periods and to quantitatively distinguish the contributions of human activities and climate variability to runoff declines [20]. Compared to other methods, process-based hydrologic models can mechanistically simulate the hydrologic cycle and have physical advantages not achieved by other statistical or empirical methods [21]. Since its appearance, the VIC model has been applied in various geographical regions and can accurately simulate complex processes in the hydrological cycle [22–24]. Meanwhile, the VIC model distributes various processes in the simulating runoff component, such as the snowmelt process, the flow process, and the permafrost process [25]. Therefore, the VIC model was chosen as the tool to quantify the impact of runoff in this study.

Recently, the Ebinur Lake basin (ELB) in Xinjiang, China, has become a hotspot for research on hydrology and water resources under changing conditions as the lake’s wetlands decline, land degradation increases, and water resource supply and demand are in conflict in recent decades [26–30]. However, few studies quantitatively distinguish between the effects of climate change and human activities on runoff and its components over a relatively long period. There is a need for further research to detail hydrologic and water management changes to better manage water resources in the ELB. Our study focuses on the responses of runoff and its components to human activities and climate change in a typical arid inland region where ER projects have been conducted in recent decades, including changes in land cover and vegetation leaf area. Our specific objectives were to (a) quantify the effects of climate change and human activities on runoff, baseflow, surface runoff, and snowmelt; (b) to compare changes in land cover, leaf area index (LAI), runoff, and their components before and after implementation of the ER plan; and (c) to reveal the uncertainty of various calibration algorithms for hydrologic model simulations.

## 2. Study area dataset

### 2.1 Study area

Our study area includes the Bortala River basin, the Jing River basin and the adjacent wetland of Lake Ebunur, and parts of the Kuitun River basin extending from 4°20’N to 45°23’N and 79°53’E to 83°53’E; the area is located in the arid inland region of northwest China [31]. The total area of the study area is 50 000 km^2^ and includes the Mongolian autonomous prefecture of Bortala and part of the city of Kuitun in the Xinjiang Uygur autonomous region, China [32]. Average July and January temperatures in the basin are about 27.3°C and -17.2°C, respectively, and average annual precipitation ranges from 89 to 169 mm, indicating a typical dry continental climate [33]. The average annual runoff in the basin from 1964 to 2017 was 4.27 × 108 m^3^, which is 62.79% of the annual summer runoff, a typical pattern for dryland runoff [28].

### 2.2 Dataset

#### 2.2.1 Meteorological data

Four meteorological variables on a daily scale from 1964 to 2017 were selected for the study area to run the VIC model. Maximum temperature, minimum temperature, precipitation, and wind speed. Includes data for 7 locations obtained from the China Meteorological Administration (CMA, http://data.cma.cn/). Because the topography of the study area is highly variable and conventional interpolation methods are unable to account for temporal and spatial variability in precipitation, a thin-slab interpolation method based on climatic averages was used, and climatic averages were obtained from WordClim [34,35] (https://www.worldclim.org/data**)**. The thin plate interpolation method provides better climate estimates by using mature climate background field data as a third variable to control for errors that can be difficult to correct during the interpolation process [36].

All the above meteorological data were interpolated to a gridded data set of 0.05° using the TPS interpolation algorithm within the WordClim climate mean. The WordClim dataset is a global gridded meteorological dataset interpolated from nearly 60,000 meteorological station data using sparse splines with two environmental covariates (elevation and distance to the coast) and MODIS-based satellite products [37]. In this study, a standardized normalized homogeneity test (SNHT) was performed on meteorological data to identify data spikes and outliers that were not due to climatic factors. Of the selected meteorological stations, only a few stations had missing values before 1970 after the SNHT and homogenization evaluation, so this did not have a significant impact on the research results. Also, since the TPS interpolation is performed under the average background meteorological field, the interpolation data do not significantly affect the research results. The rate of change of -6.5°C km^-1^ was adjusted to reflect the actual decrease in temperature with altitude, using the relationship between temperature and altitude at measured meteorological stations [38].

#### 2.2.2 Environment variables

These soil data were taken from the World Soil Database (HWSD), where soil attributes were stored in a 30 arc-second grid, and were mainly used for the parameterization of the VIC model [39].

Based on the delineation of the impact period, we used land cover, leaf area index (LAI), and shortwave albedo from three representative years (1980, 2000, and 2017) as vegetation parameters for the VIC model simulations. These land cover, LAI, and shortwave albedo data were obtained from Landsat TM imagery, AVHRR, and MODIS products [40]. After analyzing the breakpoints, we categorized these vegetation parameters as LC _1980, LC _2000, and LC _2017, which represent the early and late phases of the ER project, respectively. These two phases coincided with the introduction of the ER plan developed by the Chinese government, which is useful for researching the effects of land-use change and ecological restoration plans. To reconcile the data for LAI and shortwave albedo with the use of the VIC model, three data sets were used for different periods, each with 12 months of data. These three data sets were consistent with land cover and therefore can be used for VIC simulations under different scenarios based on the classified vegetation parameters

Topographic data were obtained from HydroSHEDS, including digital elevation models, slopes, drainage directions, flow accumulation, and river networks, respectively [41].

#### 2.2.3 Streamflow

Monthly discharges from 1964 to 1984 were used to evaluate the performance of the model, with discharges recorded by local water agencies at three hydrologic stations. Due to many missing values and heavy human interference with discharges at the Bole station, this station was not used for this model validation. The other two hydrologic stations had good flow quality and were well rated in several studies [4].

## 3. Methods

### 3.1 Breakpoint analysis

Understanding of the determinants of climate variability for regional ecological quality has primarily come from the study of hydroclimatic variables, and some scientists have used traditional trend tests or change analyses for hydroclimatic variables in the ELB [42]. However, traditional statistical testing methods are insufficient for analyzing breakpoints in nonlinear and nonstationary time series; thus, in this study, a heuristic segmentation-based algorithm was used to overcome these challenges [43,44]. The modified moving T-test concept was used to detect breakpoints in nonlinear and nonstationary time series in the heuristic segmentation algorithm [45]. The method has been used successfully to detect fractures in hydrological variables such as the Yellow and Han rivers, among others [46]. The nonstationary time series variables were first divided into stationary segments, and then the sliding pointer was moved from left to right along the time series. The statistical significance of the difference in means for two Gaussian distributed random samples μ_1_ and μ_2_ is indicated by Student’s t-test statistic.


$$t=\left|\frac{\mu_{1}-\mu_{2}}{S_{D}}\right|$$ (1)

Where

$$S_{D}={\left(\frac{(N_{1}-1)s_{1}^{2}+(N_{2}-1)s_{2}^{2}}{N_{1}-N_{2}-2}\right)}^{1/2}{\left(\frac{1}{N_{1}}+\frac{1}{N_{2}}\right)}^{1/2}$$ (2)

is the pooled variance. s_1_, s_2_ are the standard deviations of the two samples, and N_1_ and N_2_ are the number of points in the two samples.

We calculate t as a function of position in the time series and use the statistic t to quantify the difference between the mean of the time series left and right. If a larger t value means that the means in the two-time series are more likely to be significantly different, t_max_ is chosen as the cutoff value. Based on the heuristic segmentation algorithm, this study analyzed precipitation, temperature, runoff, and ET in the study area at breakpoints and then delineated different runoff influence periods in ELB.

### 3.2 Hydrological model

The VIC model is a scheme for determining the atmospheric transfer of ground vegetation, considering the water and energy balance [47]. The VIC model divides the study area into several latitudes and longitude grid cells, and in each grid, the land cover type is divided into an arbitrary number of tiles corresponding to the proportion of land cover types (e.g., grassland, cropland) in the grid cell. Runoff was simulated for each land type tile and averaged as a weighted average of the grid cells. Although the VIC model cannot accurately simulate vegetation for each period, it reflects the response of vegetation to hydrologic processes based on the climatological characteristics of the vegetation (e.g., 12-month LAI) [48].

The VIC model has been well-calibrated in numerous river basins around the world, including arid regions, and has been successfully used in water resource management, climate variability, and anthropogenic impact studies [49,50].

#### 3.2.1 Modeling setup

The ELB domain was emulated on a daily scale from 1959 to 2017 with three scenarios of VIC model strategies. Both VIC setups under the three scenarios were run at a spatial resolution of 0.05° degrees (consisting of 1794 grid cells) and on a 24-hour time scale, where each grid cell was simulated independently and could be divided into multiple vegetation heterogeneities, multiple soil types, and multiple soil types [51].

#### 3.2.2 Calibration and validation

The baseline hydrometeorological simulations were divided into two separate parts: the calibration period (1964–1974) and the validation period (1975–1984). The observed discharge data from regional hydrological stations were used for comparison with VIC-simulated discharge in the baseline period. The calibration process was mainly to optimize the infiltration, *Ds*, *Ws*, and soil depth of the VIC model [52]. We also used Differential Evolution Markov Chain (DE-MC) and shuffled complex evolution (SCE-UA) methods to optimize the parameters of the VIC model and compare the optimization performance of the two different algorithms. The effectiveness of the VIC model was described using the recommended Nash-Sutcliffe model efficiency coefficient (NSE), root mean square error (RMSE), coefficient of determination (R^2^), and percent bias (PBIAS) of the model evaluation [53]. It was assumed that the calibrated and validated VIC model from 1964 to 1984 deputized the hydrological processes of the ELB under natural conditions.

The DEMC algorithm is a new method for global optimization in the normative parameter space that combines Markov chain Monte Carlo (MCMC) simulation with differential evolution algorithms [54]. The strengths of this method include overcoming the difficulty of MCMC selecting the appropriate scale and orientation for proposal assignment and resolving the computational efficiency difficulty of reaching convergence time [55]. Relevant research has shown that DEMC can accurately and efficiently search the parameter space in the field of simulation of rainfall-runoff modeling [56]. Meanwhile, we used the traditional global parameter optimization method, SCE-UA, as a control group for the MCMC method for the VIC model parameter optimization scheme to select the optimal model parameters.

The performance of the VIC model was described by the following four indicators of statistics: NSE, RMSE, R^2^, and PBIAS and calculated by the following formulas.

$$NSE=1-\frac{\sum_{i=1}^{n}{\left({\mathrm{SIM}}_{i}-{\mathrm{OBS}}_{i}\right)}^{2}}{\sum_{i=1}^{n}{\left({\mathrm{OBS}}_{i}-\bar{OBS}\right)}^{2}}$$ (3)

$$RMSE=\frac{1}{n}\surd \overset{n}{\underset{i=1}{\sum }}{\left({\mathrm{OBS}}_{i}-{\mathrm{SIM}}_{i}\right)}^{2}$$ (4)

$$R^{2}=\frac{{\left(\sum_{i=1}^{n}({\mathrm{OBS}}_{i}-\bar{OBS})(SIM_{i}-\bar{SIM})\right)}^{2}}{\sum_{i=1}^{n}({\mathrm{OBS}}_{i}-\bar{OBS})\sum_{i=1}^{n}(SIM_{i}-\bar{SIM})}$$ (5)

$$PBIAS=\frac{\sum_{i=1}^{n}{\mathrm{SIM}}_{i}-\sum_{i=1}^{n}{\mathrm{OBS}}_{i}}{\sum_{i=1}^{n}{\mathrm{OBS}}_{i}}\times 100$$ (6)

where OBS*_i_* and SIM*_i_* are the observed and simulated values for the proceeding steps at each time, respectively; n refers to the total number of pairs of values; and and $\bar{OBS}$ are the average of the simulated and measured values, respectively. The extent of the NSE spans from -∞ to 1. The VIC simulation values were approximately close to the real state when the R^2^ and NSE were close to 1 [53]. The RMSE denotes the error of the VIC model, and the PBIAS measures the relative difference between modeled and observed values [53]. In our research, the RMSE was selected as the objective equation for the SEC-UA and DMMC calibration processes during the calibration period in this study.

### 3.3 Experimental details

#### 3.3.1 Streamflow segmentation

In this experiment, we reconstructed the streamflow and its components under different scenarios based on energy balance. No model setup for the VIC includes any lateral flow between elements or regional groundwater aquifers, so we did not include a specific groundwater component in the experiment. We partitioned the model results into surface runoff (*Q_SF_*), base flow (*Q_BF_*), and snowmelt (*Q_SM_*) based on the variable infiltration curve and the snow module [57]. Then, the main equations of the runoff and snow mode were calculated as follows:

$$Q=\overset{N+1}{\underset{n=1}{\sum }}C_{n}\times (Q_{SF}+Q_{BF})$$ (7)

$$Q_{SM}=P-\frac{dW_{e}}{dt}-p\times Q_{v}-Q_{e}$$ (8)

In addition to the runoff components described above, *C_n_* is the vegetation fractional coverage for the nth vegetation tile, *C_N+1_* is the bare soil fraction, and *Q* is the total runoff in Eq 7, where *P* is the precipitation, *Q_v_* is the sublimation from blowing snow, and *Q_e_* is the evaporation and sublimation from the snowpack, for $\frac{dW_{e}}{dt}$ is the rate of snow water accumulation, and *p* is the spatial probability of occurrence of blowing snow for a time increment *dt* in Eq 8. The total runoff was a combination of base runoff and surface runoff, excluding snowmelt. Based on this classification, we compared the seasonal runoff and its components in the above study area. We categorized the seasons like June, July, and August for summer; September, October, and November for fall; December, January, and February for winter; and March, April, and May for spring. In the current study, there were three components of total runoff: surface runoff (referring to direct surface rainfall-runoff), baseflow (referring to subsurface rainfall-runoff), and snowmelt runoff (surface runoff from melting snow).

#### 3.3.2 Detection of human activities and climate variability contributions

After the implementation of the corresponding ER project, the hydrological cycle in Northwest China has been greatly influenced, but the contributions of climate change and human activities to changes in runoff and its components are still debatable [3]. We applied the concept of scenario modeling and the classification of sensitive periods to determine the response of runoff changes to human activities and climate change. We used three scenarios: *VIC_1980* was the baseline scenario, which was used both for model calibration and validation and for reconstructing runoff from the state of nature, with the 1980 land cover and LAI data; *VIC_2000* simulates the runoff from Impact Phase Ⅰ, which was mainly used to separate direct and indirect human activities, with the 2000 land cover and LAI data; and *VIC_2017* simulates the runoff from Impact Phase Ⅱ, which serves a similar purpose as the *VIC_2000* scenario, with the 2017 land cover and LAI data (Table 1).

**Table 1:** Simulation scenarios for detecting climate variability and human activity on streamflow and its components.

Scenarios	LC	Period	LAI	Objectives
VIC_1980	Early-Restoration-1980	1964–2017	LAI-1980	Reconstruction of natural streamflow and its components during impact phase
VIC_2000	Late-Restoration-2000	1985–2000	LAI-2000	To identify the effect of human activities during the impact phase I
VIC_2017	Late-Restoration-2017	2001–2017	LAI-2017	To identify the effect of human activities during impact phase II

**Fig 1:**
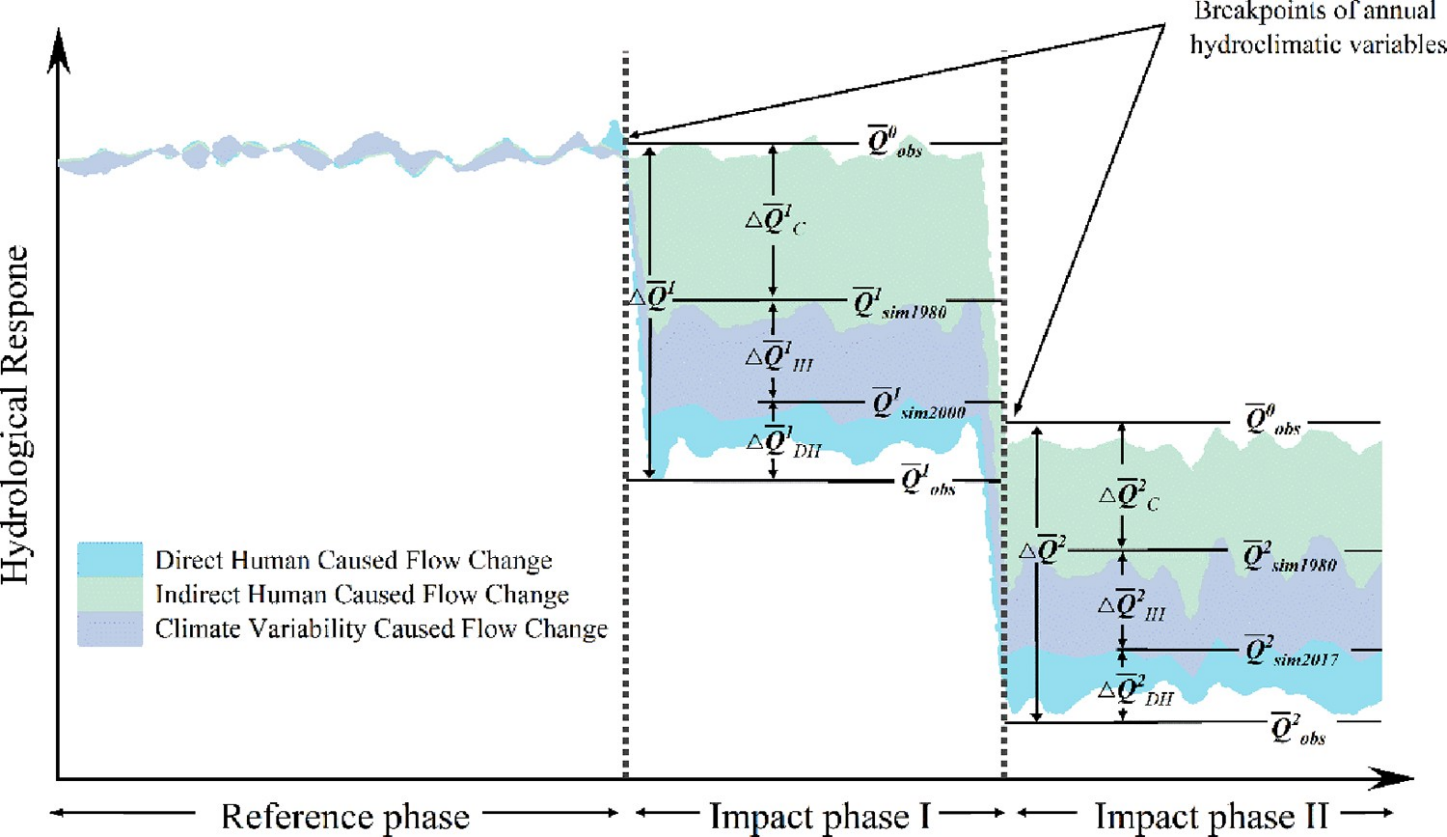
A schematic to determine the effects of climate change and human activities on streamflow change.

The historical runoff time series needs to be separated into two segments, the second representing a period (referred to herein as the impact phase) influenced by both climate variability and human activities (Fig 1), according to the following equations:

$$\Delta {\bar{Q}}^{1}={\bar{Q}}_{obs}^{1}-{\bar{Q}}_{obs}^{0}$$ (9)

$$\Delta {\bar{Q}}^{2}={\bar{Q}}_{obs}^{2}-{\bar{Q}}_{obs}^{0}$$ (10)

Where and $\Delta {\bar{Q}}^{2}$ denote the changes in a total runoff for impact phases I and II, respectively; ${\bar{Q}}_{obs}^{0}$, ${\bar{Q}}_{obs}^{1}$, and ${\bar{Q}}_{obs}^{2}$ are the observed runoff for the reference phase, impact phase I, and impact phase II, respectively.

Second, the change in runoff caused by human activities was determined by reconstructing the scenarios (Table 1) of runoff in the natural state for different impact periods by the following equations:

$$\Delta {\bar{Q}}_{H}^{1}={\bar{Q}}_{obs}^{1}-{\bar{Q}}_{sim1980}^{1}$$ (11)

$$\Delta {\bar{Q}}_{H}^{2}={\bar{Q}}_{obs}^{2}-{\bar{Q}}_{sim1980}^{2}$$ (12)

where ${\bar{Q}}_{H}^{1}$ and ${\bar{Q}}_{H}^{2}$ indicate the changes in runoff due to human activities during Impact phase 1 and Impact phase 2, respectively; ${\bar{Q}}_{sim1980}^{1}$ and ${\bar{Q}}_{sim1980}^{2}$ represent the mean runoff from the simulated impact phases I and II, respectively, under the VIC_1980 scenario.

The effects of human activities on hydrological processes can be categorized as direct or indirect, depending on whether they directly affect the water cycle. Indirect human activities consist mainly of changes in land cover properties on the land surface, such as reclamation of land and afforestation. These activities mainly affect hydrological processes on the land surface, which further affect runoff. Direct human activity is defined as all human activities that directly affect the hydrological cycle, except for land use/cover change, including dam construction and reservoir operations, human water withdrawals, and agricultural and industrial applications. According to the above scenarios (Table 1), we reduced the simulated runoff from the *VIC_2000* and *VIC_2017* scenarios by the observed runoff from impact phases I and II, respectively, to obtain the corresponding runoff changes due to indirect human activities. Below are the relevant formulas:

$$\Delta {\bar{Q}}_{IH}^{1}={\bar{Q}}_{sim2000}^{1}-{\bar{Q}}_{sim1980}^{1}$$ (13)

$$\Delta {\bar{Q}}_{IH}^{2}={\bar{Q}}_{\mathrm{sim}2017}^{2}-{\bar{Q}}_{\mathrm{sim}1980}^{2}$$ (14)

$$\Delta Q_{H}^{1,2}=\Delta Q_{IH}^{1,2}+\Delta Q_{DH}^{1,2}$$ (15)

where $\Delta Q_{H}^{1,2}$, $\Delta Q_{IH}^{1,2}$ and $\Delta Q_{DH}^{1,2}$ represent the changes in runoff due to human activity, indirect human activity, and direct human activity for impact phases I and II, respectively; ${\bar{Q}}_{\mathrm{sim}1980}^{1}$, ${\bar{Q}}_{\mathrm{sim}2000}^{2}$, ${\bar{Q}}_{\mathrm{sim}1980}^{2}$, and ${\bar{Q}}_{\mathrm{sim}2017}^{2}$ are the simulated average values of runoff for the VIC_1980, VIC_2000, and VIC_2017 scenarios in impact phases I and II, respectively.


$$\Delta Q^{1,2}=\Delta Q_{H}^{1,2}+\Delta Q_{C}^{1,2}$$ (16)

After the above scenarios and calculations, we divided the factors that contributed to the total runoff change into two parts: human activities and climate variability, although, these two parts often interact with each other in the basin hydrological cycle. Therefore, the total runoff change (Δ*Q*^1,2^)due to the combined effects of climate variability and human activities is described by the following equation:

where $\Delta Q_{H}^{1,2}$ and $\Delta Q_{C}^{1,2}$ represent the streamflow changes induced by climate variability and activities in impact phases I and II, respectively.

## 4. Results

### 4.1 Model performance evaluation

**Fig 2:**
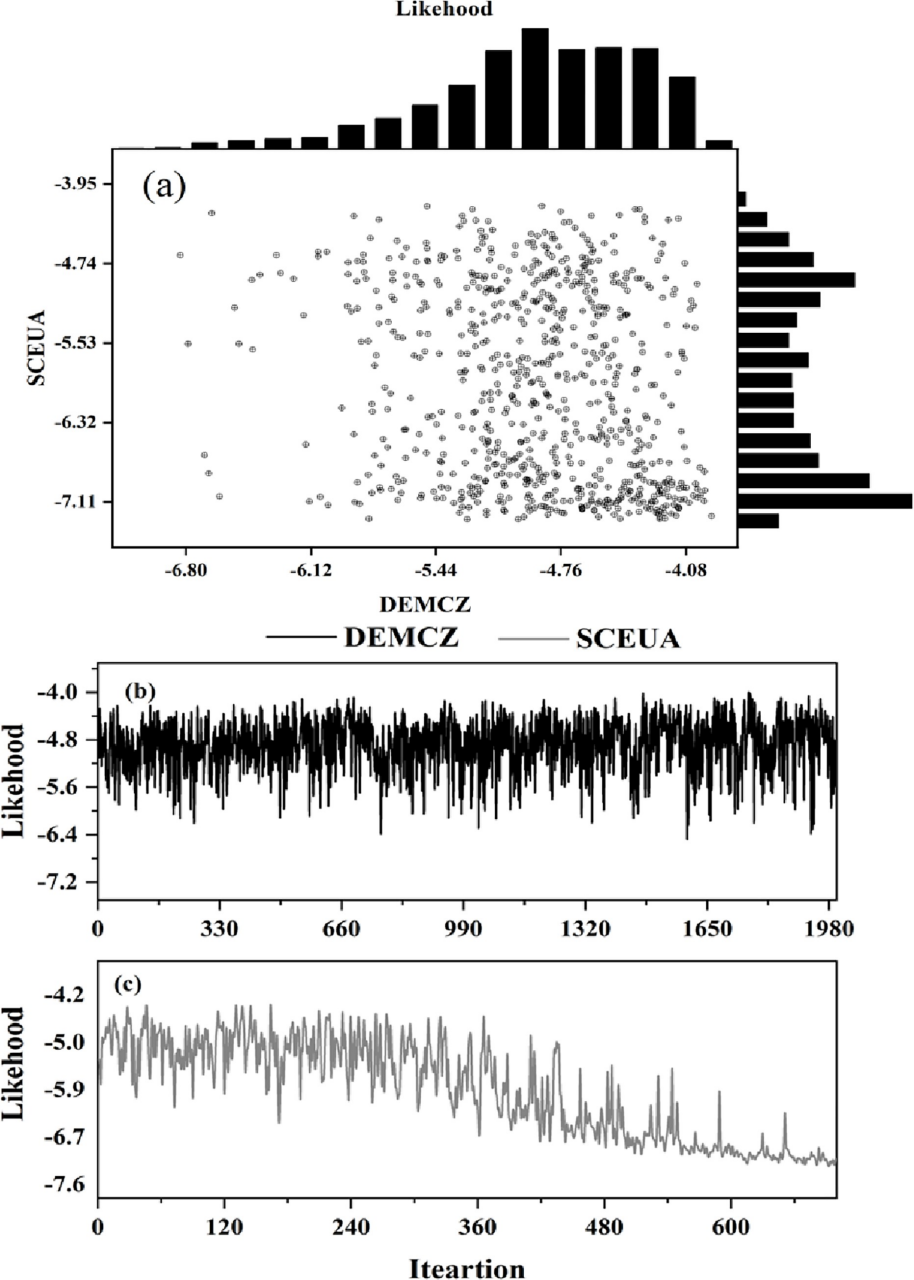
Scatterplot comparing the RMSE values of the two algorithms for the monthly emissions during the calibration period (a). Objective function trajectories for the DEMC and SC-EUA algorithms (b, c).

Based on the SCEUA and DEMC parameter calibration algorithms described in Section 3.2.2 and the observed series of measured monthly runoff from the two sites, we obtained the performance of the VIC model and the objective function trajectories of the two algorithms. All boundary conditions and input data were kept constant in this study, and two calibration algorithms were used to calibrate the VIC model for each of the five parameters. These parameters were the shape of the VIC curve (*b*), which controls the amount of water that can infiltrate into the soil; the thickness of the second and lowest soil layers *d_2_*(m) and *d_3_*(m), which affect the maximum storage capacity for transpiration; the fraction of maximum baseflow where nonlinear baseflow occurs; and the fraction of maximum soil moisture of the third soil layer (*W_s_*) where nonlinear baseflow comes from [58]. Fig 2 depicts the objective function trajectories of SC-EUA and DEMC after 2000 iterations, with Fig 2(A) depicting the scatter plot and histogram of the two algorithms, and Fig 2(B) and 2(C) depicting the objective function trajectory of the two algorithms over time. The scatter plot demonstrates that there is no correlation between the two algorithms’ trajectories. The SC-EUA objective function trajectory was mainly concentrated in the range of -6.32 to -7.11, which was more laxly distributed than the DEMC results, and the DEMC was concentrated in the low-value region below -4.76, proving that the DEMC algorithm could explore the parameter space more broadly and efficiently than the SC-EUA algorithm. According to Fig 2(B) and 2(C), as the number of iterations increases, the RMSE of SC-EUA gradually increases compared to DEMC, indicating that the DEMC algorithm can effectively reach the convergence space. The optimal calibration algorithm from DEMC outperformed the SC-EUA algorithm in both the calibration and verification periods, as shown in Table 2. According to the simulation results, the NSE of both the DEMC and SC-EUA algorithms exceeded 0.60, achieving the simulation’s reliability level, and the simulation performance of the Wenquan station was superior to that of the Jinghe station, with the NSE and R2 of the DEMC algorithm being approximately 6% greater than those of the SC-EUA.

**Table 2:** Performance evaluation of the VIC simulation.

Algorithms	Gauge station	Calibration				Validation			
		NSE	R	PBIAS	RMSE	NSE	R	PBIAS	RMSE
DEMC	Wenquan	0.71	0.82	1.32	1.23	0.81	0.74	2.54	1.16
	Jinghe	0.75	0.72	-2.52	2.85	0.78	0.83	3.85	2.15
SCEUA	Wenquan	0.68	0.73	4.06	2.22	0.65	0.78	4.46	3.61
	Jinghe	0.62	0.79	-19	2.35	0.77	0.69	6.02	3.86

N**ote:** DEMC is the differential evolution Markov chain algorithm, and SCE-UA is the shuffled complex evolution metropolis algorithm.

**Fig 3:**
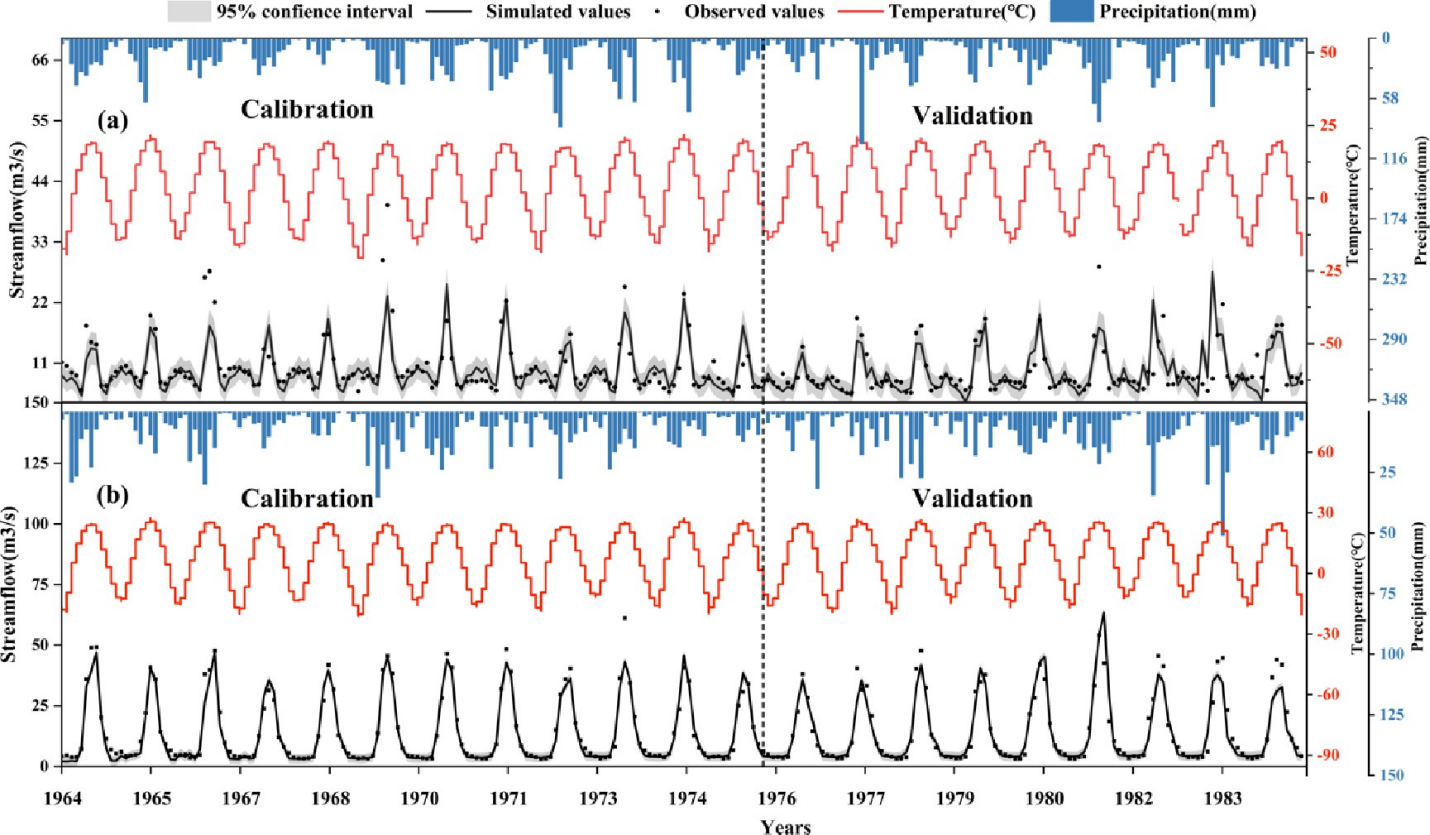
Comparison of simulated streamflow with observed streamflow from two hydrological stations, detailed model performance is shown.

Fig 3 depicts a comparison of simulated and observed runoff from two stations during the calibration and verification periods (including precipitation and temperature for the corresponding period). Although there were some over- or underestimations of streamflow at the Wenquan and Jinghe stations compared to measured streamflow at certain times, the simulation results capture the observed variation and magnitude of streamflow over monthly time well. The simulation results revealed that there was insufficient stability in the summer, and the simulated streamflow from the Wenquan station became less stable during the validation period, whereas the Jinghe station demonstrated better homeostasis throughout the simulation. As previously stated, the phenomenon of simulated underestimation may be the effect of glaciers on streamflow was not considered, and while precipitation increases in summer, more precipitation evaporates in the atmosphere, resulting in scarce water resources.

### 4.2 Breakpoint determination

**Fig 4:**
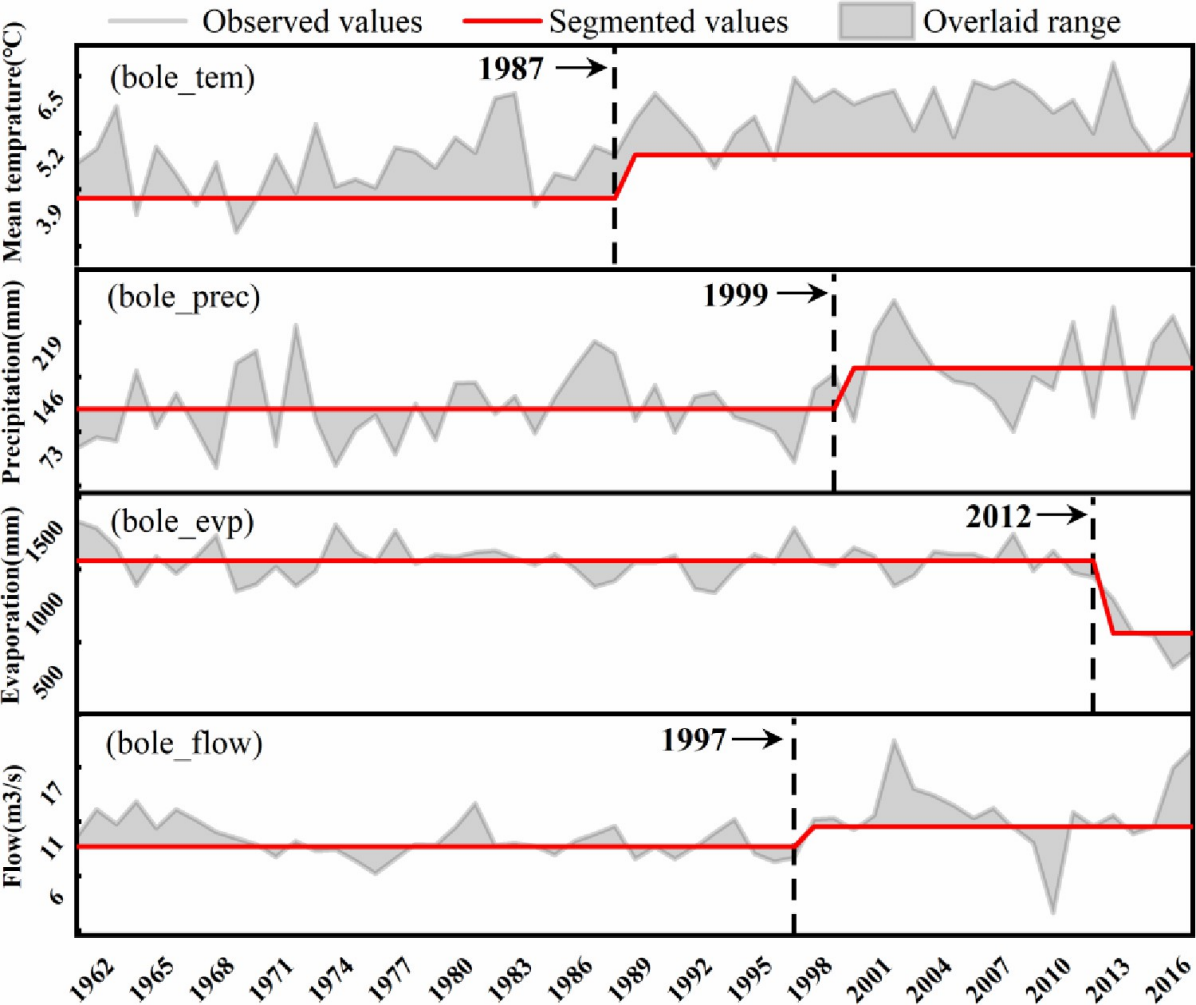
Temporal changes of hydroclimatic variables at Bole station (*p*<0.05). The gray line, red line, and gray area represent the runoff observation, breakpoint segmentation value, and coverage area, respectively.

**Fig 5:**
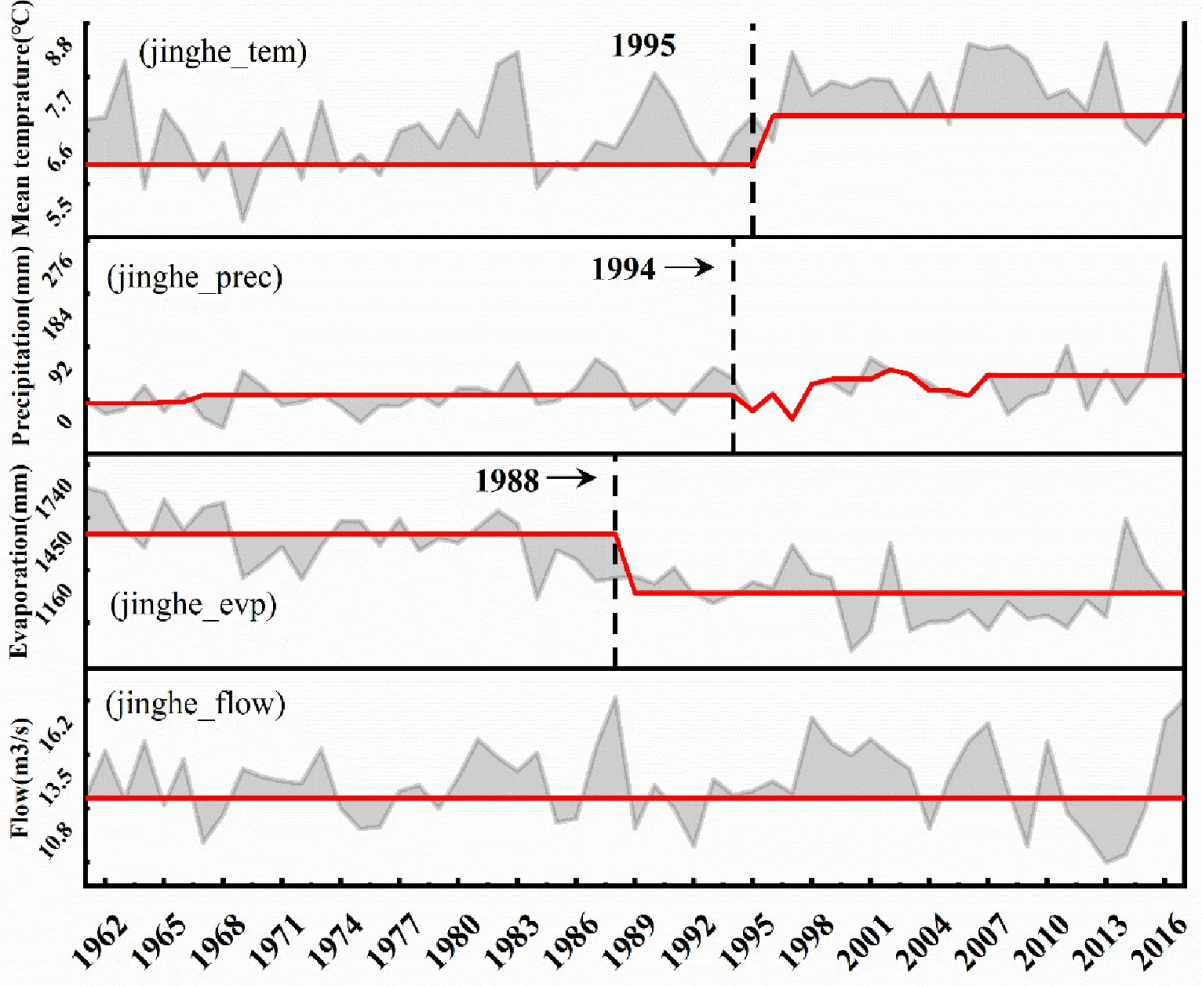
Temporal changes of hydroclimatic variables at Jinghe station (*p*<0.05). The gray line, red line, and gray area represent the runoff observation, breakpoint segmentation value, and coverage area, respectively.

**Fig 6:**
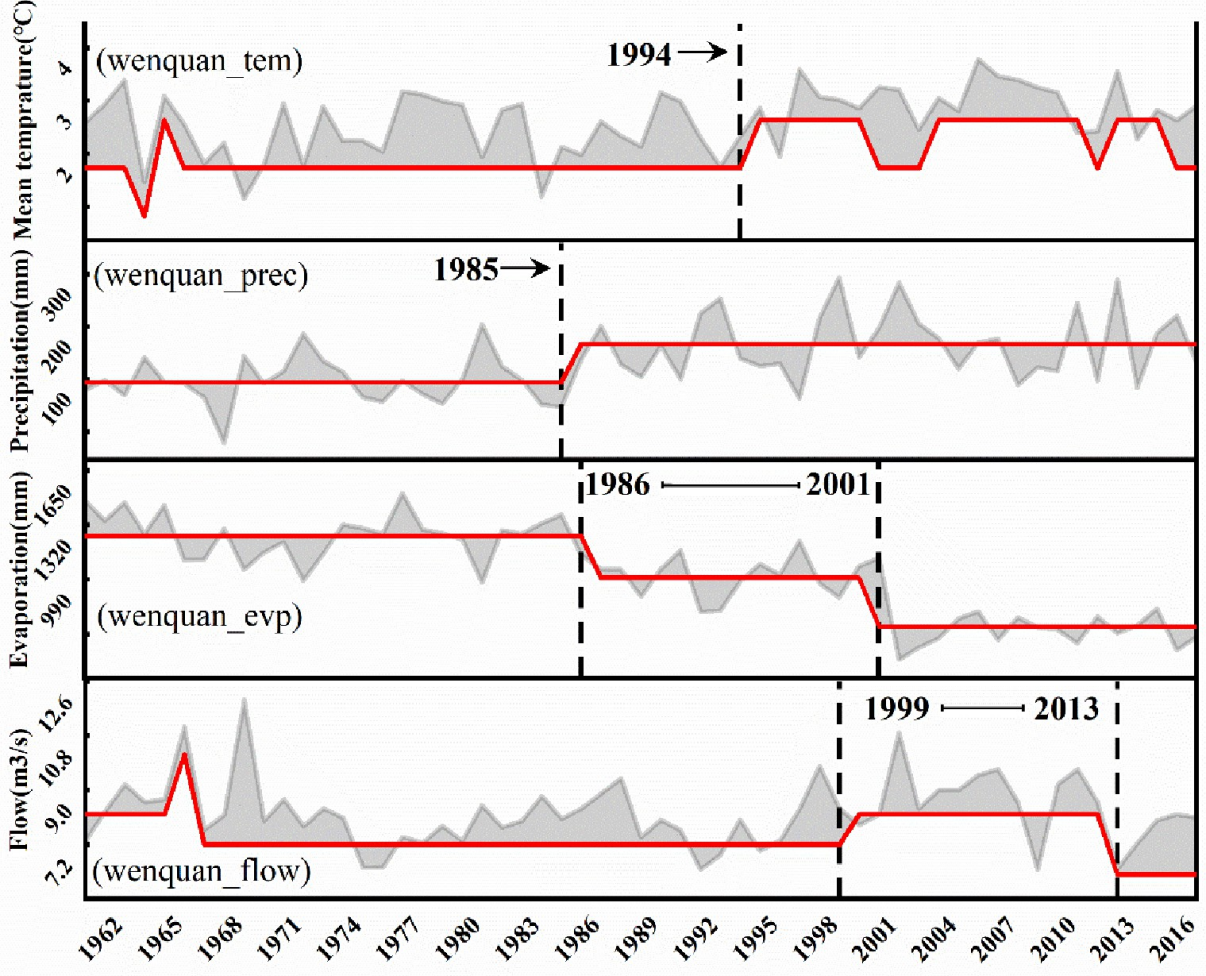
Temporal changes of hydroclimatic variables at Wenquan station (*p*<0.05). The gray line, red line, and gray area represent the runoff observation, breakpoint segmentation value, and coverage area, respectively.

The heuristic segmentation algorithm identifies abrupt changes in hydrologic variables in various regions of the ELB, which typically occur around 1985 or around 2000. Bole city’s average annual temperature increased significantly after 1987, average annual precipitation increased significantly after 1999, average annual evapotranspiration decreased significantly after 2012, and average annual streamflow increased dramatically after 1997 (Fig 4). The average annual temperature of Jinghe County increased significantly after 1995, the average annual precipitation showed a gradual upward trend after 1994, and the average annual ET decreased obviously after 1998, while the average annual streamflow did not change (Fig 5). The average annual temperature in Wenquan County exhibited a gradient change after 1994, the average annual precipitation displayed a significant upward trend after 1985, the average annual ET decreased slowly the time 1986-to 2001, and the average annual flow increased and then decreased from 1999-to 2013 (Fig 6). Through a comprehensive analysis of the abrupt time-series changes in hydrometeorological variables in different regions of the study region, the heuristic segmentation algorithm was finally used to determine two significant time ranges of changes in annual hydroclimatic variables at the three stations, one of which occurred from 1985–2000 and the other from 2001–2017. The abovementioned periods of classification coincide with the implementation of ecological restoration projects such as the Three North Protection Forest System Construction Project, the Return of Cultivated Land to Forest Project, and the Grassland Ecological Protection Subsidy Program.

**Fig 7:**
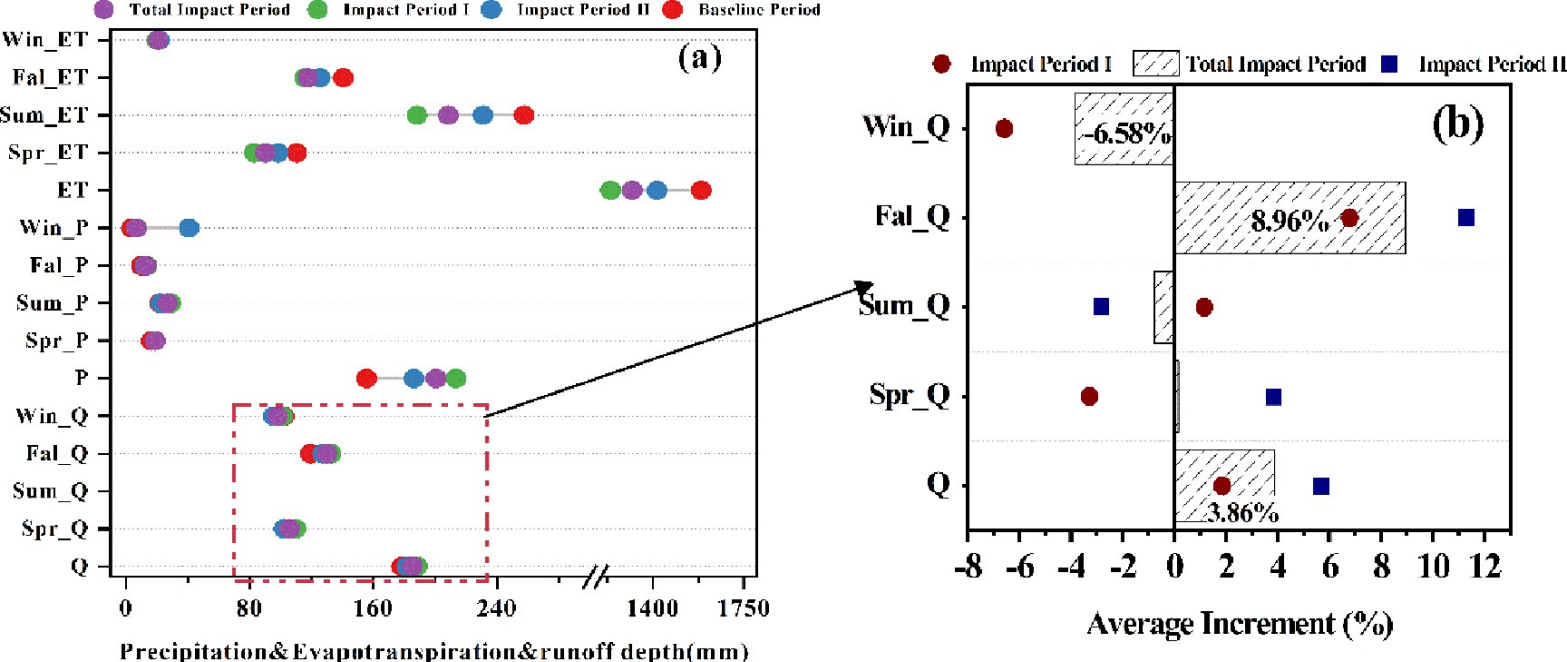
Comparison of average changes in hydroclimatic variables between the impact period and the base period. Where Win_ET, Fal_ET, Sum_ET, Spr_ET, ET, Win_P, Fal_P, Sum_ET, Spr_P, P, Win_Q, Fal_Q, Sum_Q, Spr_Q, Q represent the average evapotranspiration value in winter, the average evapotranspiration value in autumn, the average evapotranspiration value in summer, the average evapotranspiration value in spring, the average annual average evapotranspiration, winter average precipitation, fall average precipitation, summer average precipitation, spring average precipitation, annual average precipitation, winter average runoff, fall average runoff, summer average runoff, spring average runoff, annual average runoff.

As described in Subsection 3.3.2, the reference phase was assumed to be independent of human activities and climate change to facilitate a comparison with runoff during the impact period, thus distinguishing the contribution of human activities and that of climate change to runoff changes. In other words, the observed runoff was very close to its natural state. By the above-mentioned analysis, the period 1964–1984 was finally determined as the reference phase, the period 1985–2000 as impact phase Ⅰ, and the period 2001–2017 as impact phase Ⅱ. Fig 7 compares the differences in the average values of hydroclimatic variables for the reference phase and impact phases Ⅰ and Ⅱ. In general, the ET in the impact phases decreased significantly compared with the reference phase, declining from 1750 mm in the reference phase to approximately 1310 mm in impact phase Ⅰ, and the decreasing trend of summer ET was obvious, while the ET in impact phase Ⅱ was larger than that in impact phase Ⅱ. Precipitation increased by 60 mm during the whole impact phase compared with the reference phase; at the same time, the variation in winter rainfall was obvious, with impact period II being much higher than the other periods. Fig 7(B) reveals the degree of change in runoff during different impact phases compared to the reference phase, from which it could be found that runoff increased by 3.86% during the whole impact period, except for winter and summer when it showed a decreasing trend. The other seasons all increased, with the highest increase of 8.96% in autumn. The trends in the above hydrologic variables tell us that hydrologic conditions are getting better throughout the ELB; however, the question of whether this improvement is a key factor of humans’ efforts, or a selection of natural climatic factors needs to be assessed quantitatively, which we do in the following sections.

### 4.3 Land cover and LAI changes

The land cover in the ELB has undergone considerable variation in the past 40 years. Here, we used the latest land cover classification system for cropland, forest, grassland, wetland, water, snow/ice, impervious surface, and bare land because the variation in the above land cover types had an unnoticeable effect on the hydrological cycle [59]. Differences in ER plans before and after were calculated by taking 1980 as the base year and calculating the differences between 2000 and 2015 land-use data for comparison with the base year, respectively. To indicate the vegetation dynamics, we selected the monthly average LAIs from April-October of each year from 1981-to 2019.

**Fig 8:**
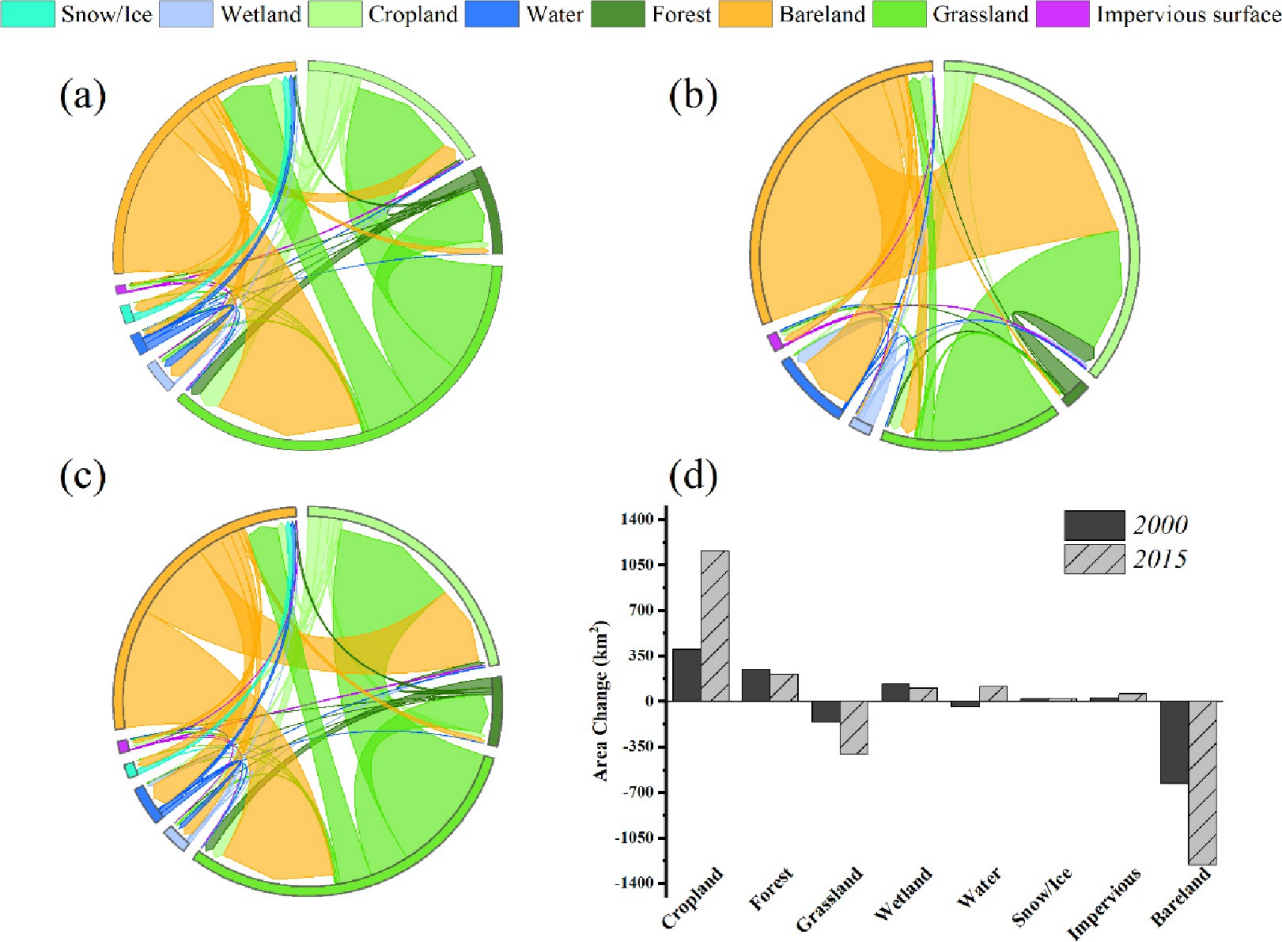
Land cover type changes from 1980–2015: (a) area shift in land cover types from 1980 to 2000; (b) area shift in land cover types from 2000–2015; (c) area shift in land use from 1980–2015; (d) changes in the area of 8 land cover types in 2000 and 2015 relative to 1980.

As Fig 8(B) shows, in addition to a significant decrease in grassland and bare land (by 1256 km^2^), all other land cover types increased, with the largest increase in cropland (by 1148 km^2^), followed by that in the forest, wetland, water, impervious surface, and permanent snow/ice. The increment in forest area may be attributed to afforestation programs, and the significant increase in cropland and impervious surfaces may be due to local population growth and economic development. By exploring the transfer pattern of each land cover type in the past 40 years, we discovered that the growth of cropland area in three different periods was mainly transferred from grassland and barren land and accompanied by a little forest, wetland, and impervious surface, in which the total transfer of bare land and grassland to cropland was 643 km^2^ from 1980–2000 (Fig 8(A)); the total transfer of the two abovementioned types was 771 km^2^ from 2000–2015 (Fig 8(B)), and the total transfer of bare land and grassland was 1336 km^2^ in the entire period (1980–2015) (Fig 8(C)). At the same time, the cropland area increased more significantly in impact period Ⅱ (2001–2017) than in impact period Ⅰ (1986–2000). The growth of forest area was transferred mainly from grassland, but the forest was also converted to grassland and cropland. In the period 1980–2000, the forest was mainly transferred to grassland (Fig 8(A)), while in the period 2000–2015, the forest was mainly transferred to cropland (Fig 8(B)), which reflects the intensification of agricultural production activities. Wetlands and water are key types reflecting local hydrologic health, as shown in Fig 8. We found an increase in the area of both wetland and water compared to 1980, except for water, which decreased from 1980-to 2000. The wetland area increased mainly by the transfer of water, grassland, and bare land, with the above three land types transferring a total of 127 km^2^ of land to wetland during the period 1980–2000 (Fig 8(A)). The trend of wetland growth decreased sharply during the period 2000–2015 (Fig 8(B)). Eventually, we determined that the permanent snow and ice cover type was also slowly increasing compared to the amount in 1980, which indicated that local hydrological resources were gradually becoming more abundant.

**Fig 9:**
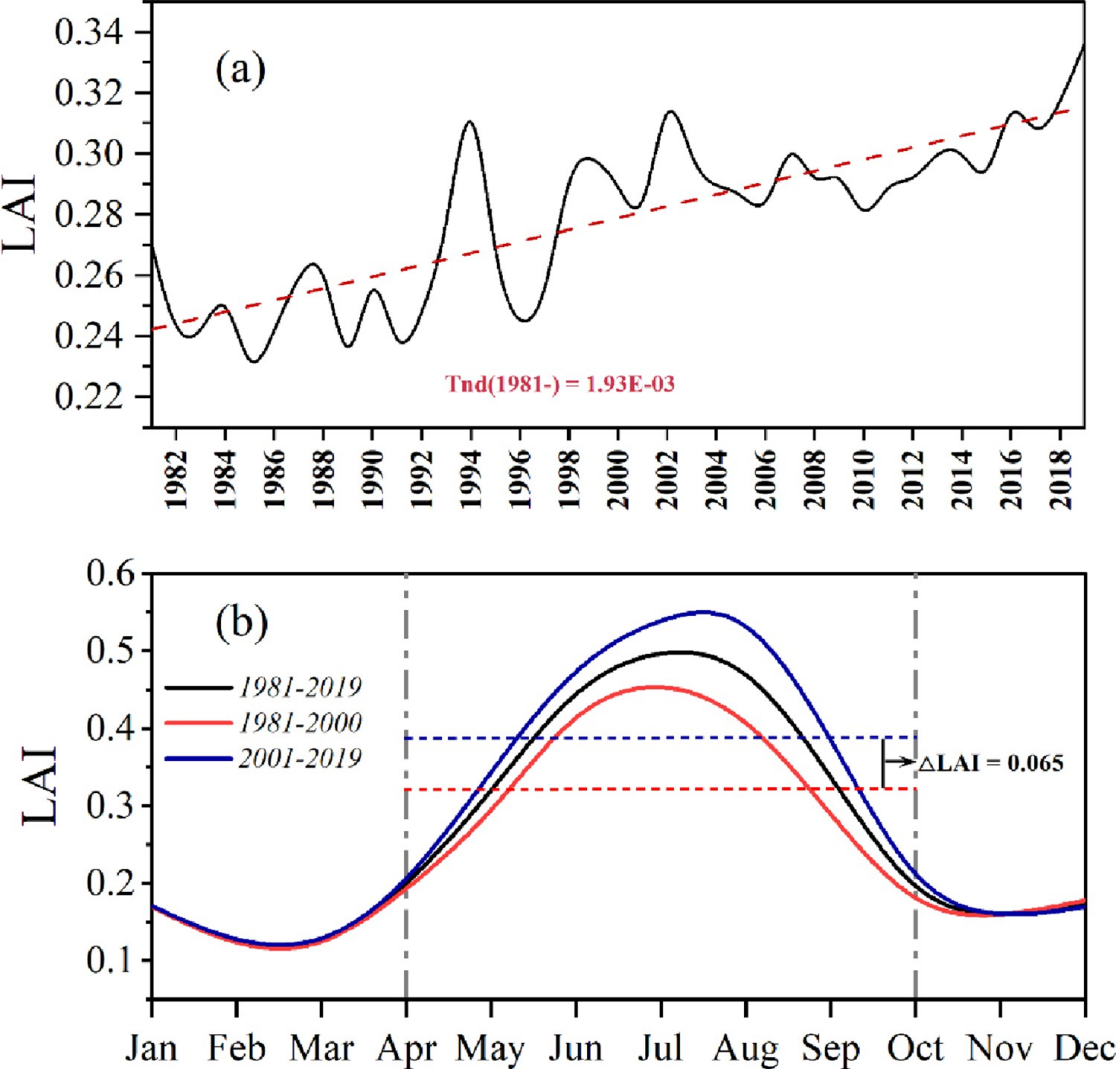
Average April-October LAI for each year from 1981-to 2019 for the entire ELB (a) and average monthly LAI for three different periods (b). The red dotted line shows the linear fit for 1981–2019, and "Tend (1981-)" denotes the trend for 19881–2019, p<0.05.

Since the ELB has undergone a continuous change in land cover type, the growth of vegetation has evolved. Fig 9 illustrates the inter-and intra-annual variation in the LAI during the vegetation growing season in the ELB from 1981-to 2019. The LAI showed an increasing trend of 0.002 per year throughout the study period, from 0.27 in 1981 to 0.34 in 2019, while the magnitude of change before 2008 was greater than that of change after 2008. Narrowing the study scale to intra-annual variation, we found that the LAI showed a trend consistent with runoff variation throughout the study period, with peaks in summer (June, July, and August) and valleys in the other periods. Dividing the intra-annual variation in the LAI into two impact periods corresponding to Subsection 4.3, we found that the LAI in impact phase Ⅰ (1981–2000) decreased by an average of 0.0064 during the vegetation growing season compared to impact phase Ⅱ (2001–2019) (Fig 9(B)).

### 4.4 Contributions analysis

This analysis was mainly concerned with the changes in runoff and its components in different impact phases corresponding to the baseline phase, along with the distinction between the effects of human and climatic factors on runoff according to the method described in Subsection 3.3.2. The interannual variation in streamflow, surface runoff, baseflow, and snowmelt was first calculated based on the difference between the designed scenario simulations and the observed runoff, and then the distribution of intra-annual runoff and its components under different scenario simulations was calculated. Finally, the contributions of human factors and climate factors to the increase in runoff and its components under different impact periods were derived.

#### 4.4.1 Temporal analysis

**Fig 10:**
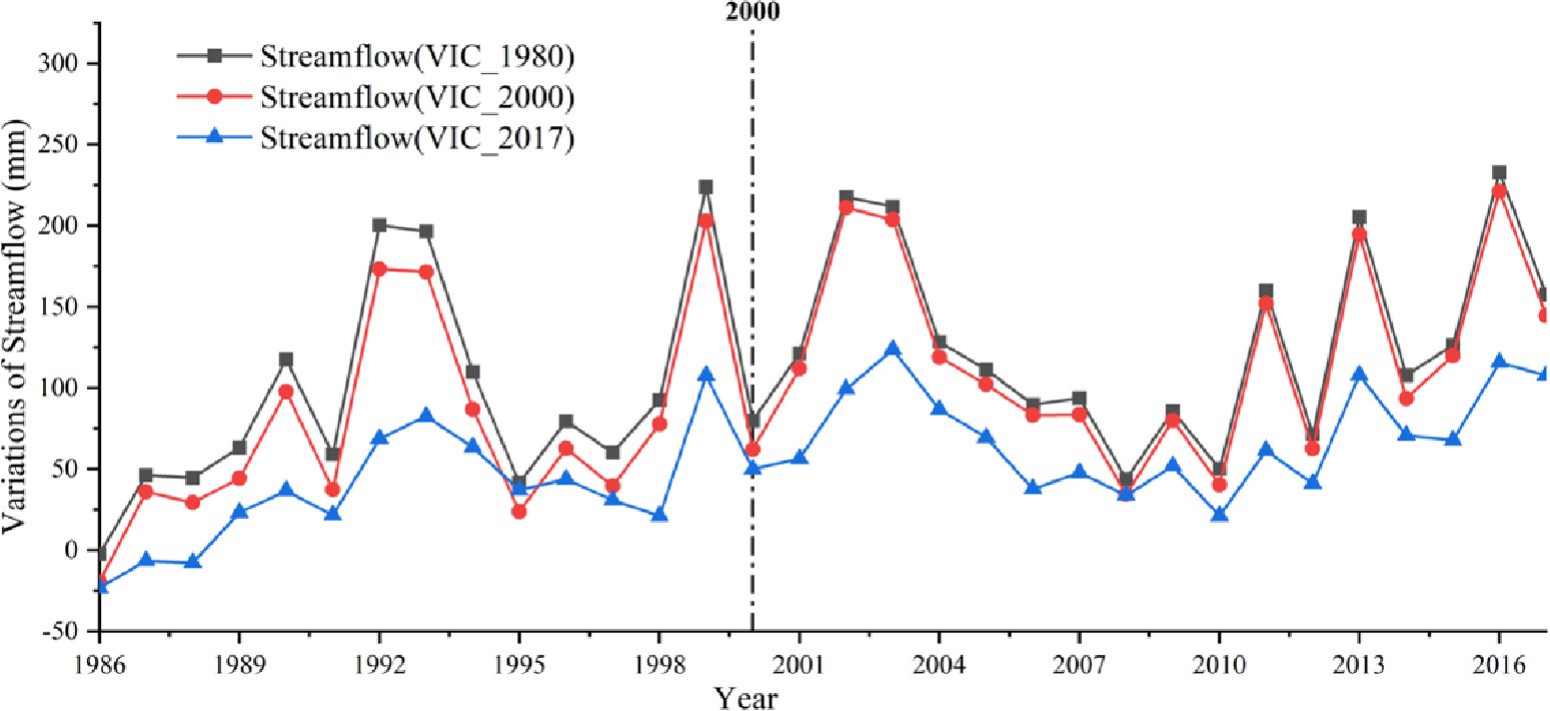
Variations in runoff depth driven by VIC_1980, VIC_2000, and VIC_2017 for the entire ELB.

The magnitude of the VIC_1980 simulated runoff variability, which represented the runoff variability under natural conditions, was considerably larger than that of the other scenarios under the three VIC scenarios mentioned above, along with a similar tendency of variations to the VIC_2000 simulated runoff (Fig 10). During 1991–1995, 1997–2004, and 2009–2017, the simulated values of the above three scenarios were significantly different from the observed runoff, while the differences were ranked in the following order: VIC_1980, VIC_2000, and VIC_2017. The above results indicated that the simulated value of the VIC_1980 scenario, which represented the natural state of runoff, had more influence on runoff change than land cover transfer, and the effect of climate change on runoff in impact phase Ⅰ was much higher than that impact phase Ⅱ.

**Fig 11:**
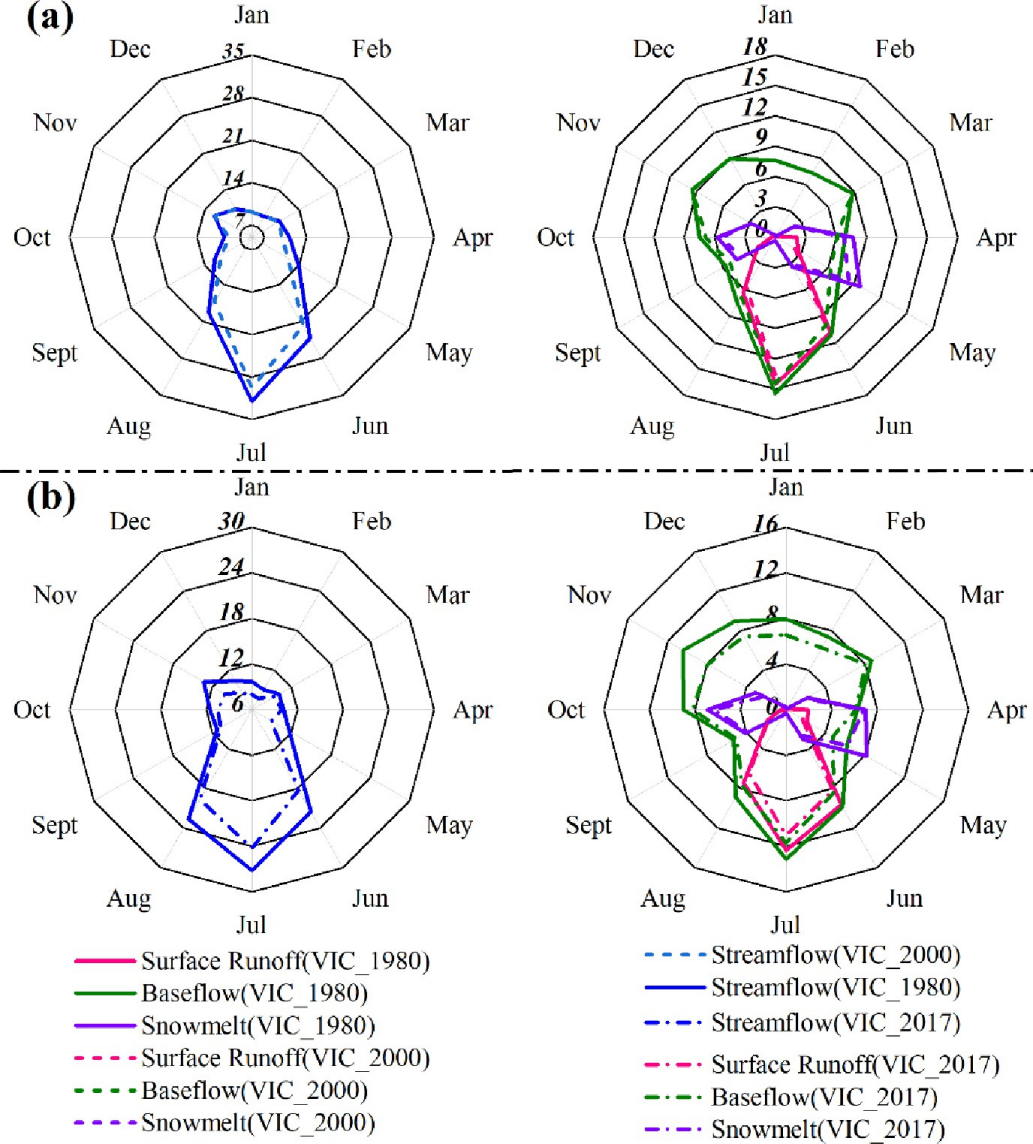
Monthly average changes in streamflow, surface runoff, baseflow, and snowmelt are driven by the VIC_1980, VIC_2000, and VIC_2017 scenarios for the entire ELB during impact phase Ⅰ (a) and impact phase Ⅱ (b).

The intra-annual trends of runoff and its components under different scenario simulations indicated obvious differences (Fig 11). The simulated runoff of VIC_1980 and VIC_2000 showed the same monthly distribution trends within impact phase Ⅰ, and high flow periods occurred in June, July, and August, which was consistent with the variation pattern of inland river runoff in the arid region. However, the simulated runoff under natural conditions (VIC_1980) was significantly higher than the VIC_2000 simulated runoff, which was due to the increase in runoff due to the gradual humidification of the regional climate. The same patterns were found for surface runoff, baseflow, and snowmelt, and the simulated runoff under natural conditions was consistent with the corresponding intra-annual distribution pattern of simulated runoff for the VIC scenario for any of the impact periods. Baseflow showed a relatively homogenous distribution compared to other runoff components during the intra-annual period, with high values occurring mainly in summer; additionally, surface runoff almost disappeared during the dry season and was concentrated in May, July, and August. Finally, snowmelt occurred mainly in spring and near the beginning of winter. A comparison of the intra-annual distribution of runoff and its components in impact phase Ⅰ and impact phase Ⅱ revealed that the average difference between simulated runoff in the natural state (VIC_1980) and the simulated runoff in VIC_2017 was higher than the average difference with the simulated runoff in VIC_2000, which was similar to the simulated pattern of interannual variation and might be caused by the inconsistency of ER plans or agricultural development in the two impact periods.

#### 4.4.2 Contributions of human activities and climate variability

**Fig 12:**
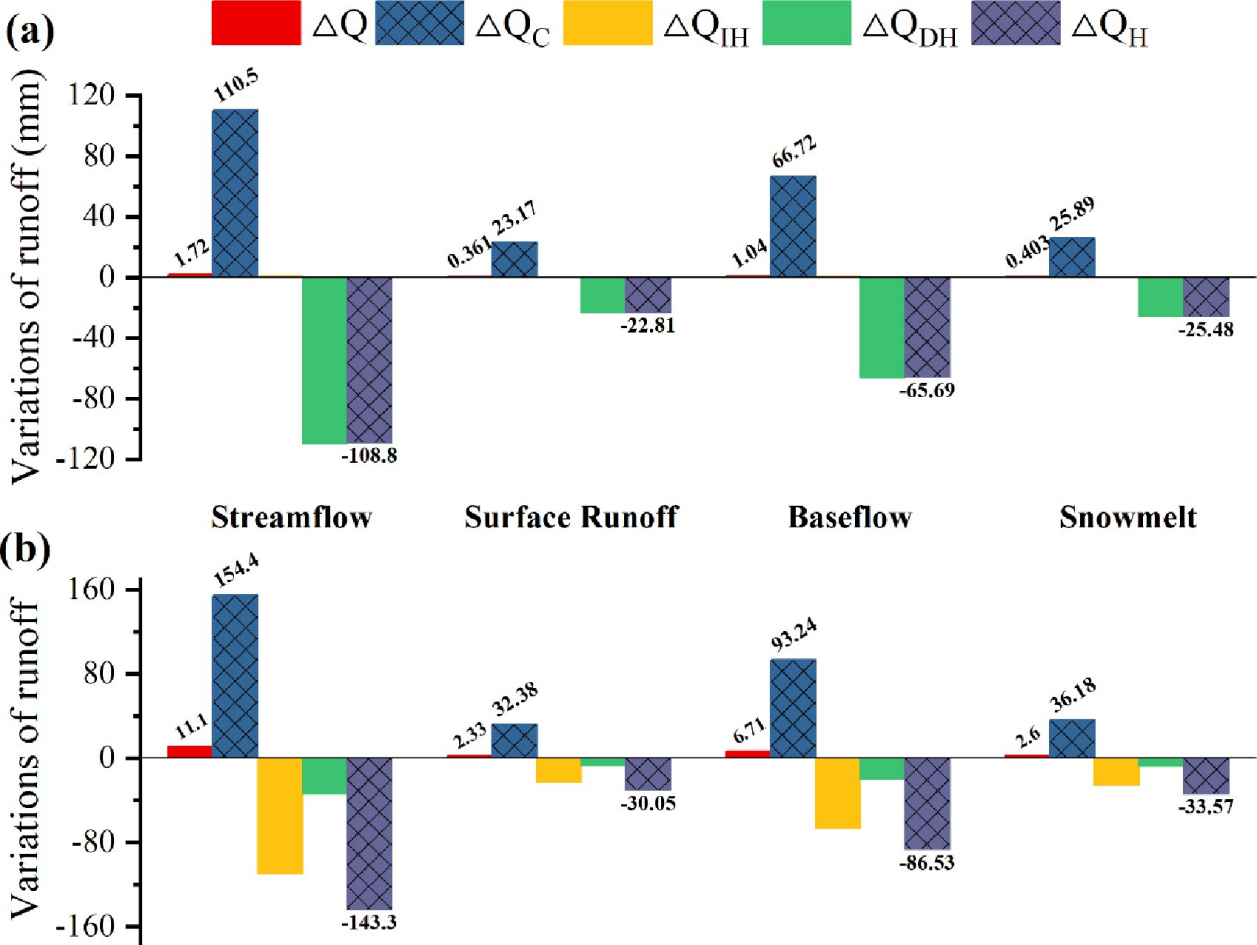
Effects of climatic and human factors on increasing trends in streamflow, surface runoff, baseflow, and snowmelt in the ELB during different impact periods, where (a) denotes impact phase Ⅰ and (b) shows impact phase Ⅱ.

Table 3 reveals the average values of runoff and its components for the VIC scenarios simulated under the reference phase and impact phases Ⅰ and Ⅱ. Compared to the reference phase, the observed runoff increased slowly during the impact phase, whereas in contrast, the VIC-simulated runoff increased more dramatically. The average runoff growth in the impact phase under the VIC_1980 scenario was the maximum compared to the reference phase because it considered only the impact of climate change on runoff, excluding the disturbance of human activities, and therefore represented the runoff change pattern under natural conditions. The difference between the runoff simulated in VIC_1980 and VIC_2000 was relatively narrow compared to the impact phase Ⅰ and reference phase, which may be because the runoff transition between this period and the base period was relatively smooth, and human activities and climate variability did not change significantly. However, the difference in runoff between the VIC_1980 and VIC_2017 simulations was higher than that between impact phase Ⅱ and the reference phase. The above phenomena are visualized in Fig 12. The observed runoff depth accumulated an increase of 1.72 mm in impact phase Ⅰ compared with the reference phase, while the observed runoff depth accumulated an increase of 11.1 mm in impact phase Ⅱ compared with the reference phase, making it much larger than the growth rate of impact phase Ⅰ.

**Table 3:** VIC-simulated values of streamflow and its components for different impact phases and scenarios.

--Variables (mm)	Reference phase	Impact phase I	Impact phase II
Streamflow (o*bs*)	138.61	140.33	149.73
Surface runoff (o*bs*)	29.06	29.42	31.39
Baseflow (o*bs*)	83.68	84.72	90.40
Snowmelt (ob*s*)	32.47	32.87	35.07
Streamflow (*VIC_1980*)	142.62	249.13	293.06
Surface runoff (*VIC_1980*)	29.90	52.23	61.44
Baseflow (*VIC_1980*)	86.10	150.41	176.93
Snowmelt (*VIC_1980*)	33.40	58.35	68.64
Streamflow (*VIC_2000*)	-	250.04	-
Surface runoff (*VIC_2000*)	-	52.42	-
Baseflow (*VIC_2000*)	-	150.96	-
Snowmelt (*VIC_2000*)	-	58.57	-
Streamflow (*VIC_2017*)	-	-	183.39
Surface runoff (*VIC_2017*)	-	-	38.45
Baseflow (*VIC_2017*)	-	-	110.72
Snowmelt (*VIC_2017*)	-	-	42.96

According to the analytical framework in Subsection 3.3.2, the effects of climate variability and human activities on the increase in runoff and its components were categorized into different impact phases (Fig 12). During impact phase Ⅰ (1986–2000), the runoff depth cumulatively increased by 1.72 mm relative to the reference phase (1964–1985), and climate change caused a cumulative increase in runoff depth of 110.50 mm, but direct human activities caused a reduction in runoff depth of 108.80 mm. Among them, indirect human activities caused an increase in runoff depth of 0.91 mm, and direct human activities caused a decrease in runoff depth of 109.71 mm. Therefore, in general, human activities had a negative effect on the trend of runoff growth. Surface runoff, baseflow, and snowmelt increased by 0.36 mm, 1.04 mm, and 0.40 mm, respectively, relative to the reference phase (1964–1985), consistent with the trend of total runoff, and all increased due to climate change but decreased due to direct human activities. During impact phase II (1986–2000), the runoff depth cumulatively increased by 11.10 mm relative to the reference phase (1964–1985), and climate change caused a cumulative increase in runoff depth of 154.40 mm, while direct human activities caused a reduction in runoff depth of 143.30 mm. Among them, indirect human activities caused a 109.66 mm reduction in runoff depth, and direct human activities caused a 33.66 mm reduction in runoff depth; thus, the reduction in runoff depth caused by indirect human activities was higher than that caused by direct human activities compared with the impact phase Ⅰ. It was noteworthy that indirect human activities were attributed to the increase in runoff during impact phase Ⅰ compared to the reference phase (1964–1985), but indirect human activities were attributed to the decrease in runoff during impact phase Ⅱ. As mentioned above, climate variability was the main factor leading to the increase in runoff and its components in the ELB region, and human activities were the main factor leading to the decrease in runoff and its components, although in general, the increase in runoff due to climate change was greater than the decrease in runoff due to human activities; therefore, the runoff showed an increasing trend compared with the reference phase (1964–1985). Meanwhile, compared to the baseline period (1964–1985), runoff and its components were higher in impact phase Ⅱ than in impact phase Ⅰ, and the increase in runoff due to climate variability was greater than that in impact phase Ⅰ. Indirect human activities contributed to the increase in runoff in impact phase Ⅰ but contributed to the decrease in runoff in impact phase Ⅱ.

## 5. Discussion

### 5.1 The driving force behind changing streamflow

Our study traced variations in the hydrological cycle of the ELB based on VIC simulations, which were largely derived from climate change and human activities. Against the background of global warming, precipitation, and runoff in the ELB region have shown increasing trends since the 1990s, but the changes in Lake Ebinur, the ultimate catchment of the river, have shown the reverse trend. The surface area of Lake Ebinur showed an increasing trend before 2003; thereafter, it showed a rapid decline from 885 km^2^ in 2003 to 392 km^2^ in 2015 [28]. This reduction was because from 2003 to 2015, the gross regional product of the two major cities in the ELB region, Bole city, and Jinghe County, increased by 11.210 billion yuan and 5.516 billion yuan, respectively, while the area of sown crops increased by 34,700 hectares and 6.58 hectares, respectively [60,61]. These dramatic economic activities consumed many water resources in the ELB. This pattern is like the pattern presented in Fig 12, where human activities consumed most of the water resources compared to the baseline period, although there was a slight increasing trend in runoff during the impact period. The ELB and even the arid region are gradually becoming wetter due to climate change. Li, Chen found that the runoff of rivers, which were heavily recharged by snow and glaciers, showed a significant upward trend due to the increase in glacier and snow meltwater caused by the increasing temperature and precipitation each year [10]. The fragile hydrological environment of inland river basins in arid regions is particularly sensitive to human activities, such as the expansion of cultivated land and the construction of hydraulic projects [62].

Several recent studies have shown that changes in vegetation dynamics may have significant impacts on the local hydrological cycle. Zhao, A compared the records of total terrestrial water storage depletion before and after ER through gravity satellites and government reports and found a significant increase (p<0.0001) in the total terrestrial water storage depletion after ER, indicating that ER was the main cause of water depletion [63]. Xie, Liang found that the Three Northern Protection Forests Program and climate change caused an upward trend in precipitation and runoff in the arid region of Northwest China but a downward trend in the semiarid region [3]. A review of ELB hydroelectric construction showed that in approximately 2000, a total of 12 hydroelectric facilities of diverse scales were built in the middle and upper reaches of ELB rivers, mainly for irrigation and industrial water supply, as well as for flood control and electricity generation. In parallel, Wang, Liu found that the hydrological and water resource variations in the ELB region over the past 50 years were mainly due to the continuous expansion of cropland and oases, the continuous human growth and the construction of hydraulic engineering, which coincided with the pattern found in Fig 12 of this paper [4]. The above events and research were closely related to the direct human activities that led to the decline in runoff shown in Fig 12(A). Hence, for arid regions, suitable ER programs can contribute to increasing the positive feedback effect of the hydrological cycle, but the intensification of human activities is the main element of water stress in arid regions.

### 5.2 Potential limitations

It was evident from the NSE of the flow that the VIC model performed well at the Wenquan station (Table 2). However, because of the scarcity of hydrological gauging station data, the simulated runoff could hardly fully reflect the real hydrological variability in the reference phase. To remedy the abovementioned defects, this study introduced the traditional SCE-UA and DEMC algorithms from the model calibration algorithm to compare and improve the model calibration parameters, thereby better reflecting the actual hydrological pattern. The proposed results showed that the DEMC improved the NSE by nearly 0.2 compared with the SCE-UA algorithm, proving that Bayesian methods, especially the latest DEMC algorithm, could effectively improve the simulation accuracy of hydrological models. More measured hydrological datasets need to be obtained in the future, or metrics that more accurately reflect simulation accuracy need to be used. For example, Bennett, Nijssen used a complex information network model (transfer entropy) to measure differences in fluxes within hydrological models in various climate zones, and the authors used traditional correlation metrics for the analysis and found that transfer entropy provided a complimentary account of model behavior [64].

The current version of the VIC model on the runoff partitioning equation needs to be further updated, and the effect of permanent glaciers on the ELB hydrological cycle was not considered in this study because the proportion of permanent glaciers was only 0.30%. In our study, three different land cover and vegetation leaf area datasets (i.e., LC_1980&LAI_1980, LC_2000&LAI_2000, LC_2017&LAI_2017) were applied to represent the land cover and vegetation changes. Such scenarios were set up mainly to distinguish the effects of human activities and climate change on runoff changes compared to the baseline period; however, the effects of continuous changes in land cover and vegetation were not fully captured. Thus, the contribution of ER to the hydrological cycle may be underestimated to some extent. There is a need to update the VIC model in the future so that it can be better adapted to simulations under dynamic land cover and climate vegetation change scenarios. The relevant parameters can be obtained from remote sensing data in the future to update the vegetation- and land cover-related information in the VIC model and provide a more realistic level of simulation. Furthermore, human activities (irrigation, water project construction, etc.) need to be reflected in future modeling.

There was another limitation of this study that the interpolation algorithm led to uncertainty in the climate-forcing driven data [65]. Although the interpolation of the averaged climatological state (WorldClim)-based thin-slab spline functions were used, sparse meteorological station material still introduces significant uncertainty into the simulations [66]. We note that the use of gridded meteorological assimilation data in the hydrological model is effective in reducing the simulation uncertainty [67]. Therefore, different meteorological forcing datasets and interpolation methods should be compared in future studies to reduce the uncertainty in hydrological simulations. Furthermore, in reality, climate change and human activities often combine to contribute to the hydrological cycle [68]. In this study, it was difficult to use dynamic land cover and vegetation condition scenarios to reflect the complex interrelationships among vegetation, climate, and land cover, and the ultimate results were inevitably biased to a certain extent. Consequently, considering the effects of either variable individually was not necessarily accurate, and new techniques are needed to evaluate the quantitative response of the hydrologic system to both variables in an integrated approach. An improved VIC model by coupling dynamic surface parameters and meteorological parameters will facilitate the feedback of hydrological systems in complicated changing circumstances in response to this situation.

In addition, this study lacks a detailed delineation of climate change factors. In future studies, the impact of extreme drought or precipitation on runoff should be strengthened. With global warming, extreme precipitation and drought events are becoming more frequent [69–71]. These events significantly alter precipitation-runoff patterns in arid regions and thus strongly affect the local meteorological-hydrological cycle [72]. The overall results of this study, which quantified the effects of human activities and climate change on runoff, showed that climate change has a positive feedback effect on increasing runoff and human activities have a negative feedback effect on decreasing runoff. The effects of climate change on runoff growth are primarily due to a substantial increase in runoff accumulation from glacial snowpacks and altered precipitation patterns in the arid Northwest because of global warming. Central Asia was shown to have large increases in mean annual precipitation under all scenarios associated with rapid global warming at the end of the 21st century under all common socioeconomic pathways and representative concentrated pathways (SSP1-2.6, SSP2-4.5, SSP3-7.0, and SSP5-8.5) in four combinations of scenarios [73]. The impact of human activities on the decline of runoff is mainly due to the rapid decline of available water resources in the region due to population growth and economic development in recent years [74,75]. Therefore, a strict water resource management system is needed in the future to control economic water expenditures and avoid unnecessary water consumption.

## 6 Conclusion

The hydrological cycle was significantly altered by the complex circumstances in the ELB area. With VIC model scenario simulations, we were able to detect changes in runoff and its components before and after the ER plan and to quantitatively assess the main driving forces behind the support. The following main conclusions were drawn:

1. The reference period (1984–1985), impact period I (1986–2000), and impact period II (2001–2017) were identified. The increase in cropland area was mainly caused by grassland and wasteland, while the increase in forest area was mainly caused by grassland. The LAI of vegetation growth period showed a trend of 0.002 steps per year.
2. The model parameters were calibrated using the DEMC algorithm and the SCE-UA algorithm, and the results showed that the NSE of the DEMC algorithm was improved by 20% compared to the SCE-UA algorithm. The surface runoff, baseflow, and snowmelt runoff in the reference phase accounted for 20%, 60%, and 20% of the total runoff, respectively.
3. Under the three VIC simulation scenarios, the VIC_1980 simulated runoff, which represented the natural state, was higher than the other scenarios on both intra- and interannual time scales, while the average difference between the VIC_1980 simulated runoff and the VIC_2017 simulated runoff was higher than the average difference with the VIC_2000 simulated runoff, which was like the simulated interannual variation pattern.
4. Compared to the reference period, the observed runoff depth increased by 1.72 mm in impact period I and decreased by 108.80 mm due to direct human activities, while the runoff depth cumulatively increased by 110.50 mm due to climate change. In the impact period II (2001–2017), the runoff depth increased by 11.10 mm and the cumulative increase in runoff due to climate change was 154.40 mm, while indirect human activities caused a decrease in runoff of 143.30 mm. In summary, climate change was the main reason for the increase in runoff and its components in the ELB area, while human activities were the main factor for the decrease in runoff and its components.

In the ELB and even in the arid area, it is particularly necessary to implement effective ER programs that can increase the positive feedback effects of climate change and maintain the sustainability of water resources. However, ER plans implemented in arid regions should be adapted to the local ecological environment by planting crops that are suitable for the local climate and drought and salinity tolerance to reduce the consumption of total terrestrial water reserves.
